# Supplementary material for: Unveiling the Bioactive Potential of the Invasive Jellyfish Phyllorhiza punctata Through Integrative Transcriptomic and Proteomic Analyses
Source: Biomolecules. 2025 Aug 4;15(8):1121. doi: 10.3390/biom15081121 (PMC12383608; doi:10.3390/biom15081121)
Supplement: Supplementary file 1 [file biomolecules-15-01121-s001.zip › Suplementary Material/FileS3/PEPFOLD4/XP_065055878_6649/clusters.html]

 PEP-FOLD clusters


Download archive of all models  here   

|  |  |  |
| --- | --- | --- |
| # | file | sOPEP |
| # cluster 1 - Size: 1 |  |  |
|  | XP\_065055878\_6649-model1 | -7.61944 |
| # cluster 2 - Size: 26 |  |  |
|  | XP\_065055878\_6649-model2 | -7.58393 |
|  | XP\_065055878\_6649-model2.1 | -7.58393 |
|  | XP\_065055878\_6649-model2.2 | -7.58393 |
|  | XP\_065055878\_6649-model2.3 | -7.58393 |
|  | XP\_065055878\_6649-model2.4 | -7.47353 |
|  | XP\_065055878\_6649-model2.5 | -7.47353 |
|  | XP\_065055878\_6649-model2.6 | -7.42739 |
|  | XP\_065055878\_6649-model2.7 | -7.42739 |
|  | XP\_065055878\_6649-model2.8 | -7.42739 |
|  | XP\_065055878\_6649-model2.9 | -7.42739 |
|  | XP\_065055878\_6649-model2.10 | -7.42739 |
|  | XP\_065055878\_6649-model2.11 | -7.42739 |
|  | XP\_065055878\_6649-model2.12 | -7.42739 |
|  | XP\_065055878\_6649-model2.13 | -7.42739 |
|  | XP\_065055878\_6649-model2.14 | -7.42739 |
|  | XP\_065055878\_6649-model2.15 | -7.42739 |
|  | XP\_065055878\_6649-model2.16 | -7.42739 |
|  | XP\_065055878\_6649-model2.17 | -7.42739 |
|  | XP\_065055878\_6649-model2.18 | -7.42739 |
|  | XP\_065055878\_6649-model2.19 | -7.42739 |
|  | XP\_065055878\_6649-model2.20 | -7.42739 |
|  | XP\_065055878\_6649-model2.21 | -7.42739 |
|  | XP\_065055878\_6649-model2.22 | -7.42739 |
|  | XP\_065055878\_6649-model2.23 | -7.42739 |
|  | XP\_065055878\_6649-model2.24 | -7.42739 |
|  | XP\_065055878\_6649-model2.25 | -7.42739 |
| # cluster 3 - Size: 9 |  |  |
|  | XP\_065055878\_6649-model3 | -7.57981 |
|  | XP\_065055878\_6649-model3.1 | -7.57981 |
|  | XP\_065055878\_6649-model3.2 | -7.57981 |
|  | XP\_065055878\_6649-model3.3 | -7.57981 |
|  | XP\_065055878\_6649-model3.4 | -7.34272 |
|  | XP\_065055878\_6649-model3.5 | -7.34272 |
|  | XP\_065055878\_6649-model3.6 | -7.34272 |
|  | XP\_065055878\_6649-model3.7 | -7.34272 |
|  | XP\_065055878\_6649-model3.8 | -7.34272 |
| # cluster 4 - Size: 15 |  |  |
|  | XP\_065055878\_6649-model4 | -7.54103 |
|  | XP\_065055878\_6649-model4.1 | -7.54103 |
|  | XP\_065055878\_6649-model4.2 | -7.54103 |
|  | XP\_065055878\_6649-model4.3 | -7.44948 |
|  | XP\_065055878\_6649-model4.4 | -7.44948 |
|  | XP\_065055878\_6649-model4.5 | -7.44948 |
|  | XP\_065055878\_6649-model4.6 | -7.44948 |
|  | XP\_065055878\_6649-model4.7 | -7.44948 |
|  | XP\_065055878\_6649-model4.8 | -7.44948 |
|  | XP\_065055878\_6649-model4.9 | -7.44948 |
|  | XP\_065055878\_6649-model4.10 | -7.44948 |
|  | XP\_065055878\_6649-model4.11 | -7.44678 |
|  | XP\_065055878\_6649-model4.12 | -7.44678 |
|  | XP\_065055878\_6649-model4.13 | -7.44678 |
|  | XP\_065055878\_6649-model4.14 | -7.44678 |
| # cluster 5 - Size: 20 |  |  |
|  | XP\_065055878\_6649-model5 | -7.52783 |
|  | XP\_065055878\_6649-model5.1 | -7.52783 |
|  | XP\_065055878\_6649-model5.2 | -7.52783 |
|  | XP\_065055878\_6649-model5.3 | -7.52783 |
|  | XP\_065055878\_6649-model5.4 | -7.52783 |
|  | XP\_065055878\_6649-model5.5 | -7.52783 |
|  | XP\_065055878\_6649-model5.6 | -7.52783 |
|  | XP\_065055878\_6649-model5.7 | -7.52783 |
|  | XP\_065055878\_6649-model5.8 | -7.52783 |
|  | XP\_065055878\_6649-model5.9 | -7.52783 |
|  | XP\_065055878\_6649-model5.10 | -7.52783 |
|  | XP\_065055878\_6649-model5.11 | -7.52783 |
|  | XP\_065055878\_6649-model5.12 | -7.52783 |
|  | XP\_065055878\_6649-model5.13 | -7.52783 |
|  | XP\_065055878\_6649-model5.14 | -7.52783 |
|  | XP\_065055878\_6649-model5.15 | -7.52783 |
|  | XP\_065055878\_6649-model5.16 | -7.52783 |
|  | XP\_065055878\_6649-model5.17 | -7.42953 |
|  | XP\_065055878\_6649-model5.18 | -7.42953 |
|  | XP\_065055878\_6649-model5.19 | -7.42953 |
| # cluster 6 - Size: 6 |  |  |
|  | XP\_065055878\_6649-model6 | -7.47336 |
|  | XP\_065055878\_6649-model6.1 | -7.47336 |
|  | XP\_065055878\_6649-model6.2 | -7.47336 |
|  | XP\_065055878\_6649-model6.3 | -7.47336 |
|  | XP\_065055878\_6649-model6.4 | -7.47336 |
|  | XP\_065055878\_6649-model6.5 | -7.32389 |
| # cluster 7 - Size: 13 |  |  |
|  | XP\_065055878\_6649-model7 | -7.42162 |
|  | XP\_065055878\_6649-model7.1 | -7.42162 |
|  | XP\_065055878\_6649-model7.2 | -7.42162 |
|  | XP\_065055878\_6649-model7.3 | -7.42162 |
|  | XP\_065055878\_6649-model7.4 | -7.42162 |
|  | XP\_065055878\_6649-model7.5 | -7.42162 |
|  | XP\_065055878\_6649-model7.6 | -7.42162 |
|  | XP\_065055878\_6649-model7.7 | -7.42162 |
|  | XP\_065055878\_6649-model7.8 | -7.42162 |
|  | XP\_065055878\_6649-model7.9 | -7.42162 |
|  | XP\_065055878\_6649-model7.10 | -7.42162 |
|  | XP\_065055878\_6649-model7.11 | -7.42162 |
|  | XP\_065055878\_6649-model7.12 | -7.41646 |
| # cluster 8 - Size: 4 |  |  |
|  | XP\_065055878\_6649-model8 | -7.41474 |
|  | XP\_065055878\_6649-model8.1 | -7.41474 |
|  | XP\_065055878\_6649-model8.2 | -7.41396 |
|  | XP\_065055878\_6649-model8.3 | -7.41396 |
| # cluster 9 - Size: 2 |  |  |
|  | XP\_065055878\_6649-model9 | -7.3992 |
|  | XP\_065055878\_6649-model9.1 | -7.3992 |
| # cluster 10 - Size: 4 |  |  |
|  | XP\_065055878\_6649-model10 | -7.37354 |
|  | XP\_065055878\_6649-model10.1 | -7.37354 |
|  | XP\_065055878\_6649-model10.2 | -7.37354 |
|  | XP\_065055878\_6649-model10.3 | -7.37354 |
|  |  |  |
